# Graphene Oxide Nanoparticles for Photothermal Treatment of Hepatocellular Carcinoma Using Low-Intensity Femtosecond Laser Irradiation

**DOI:** 10.3390/molecules29235650

**Published:** 2024-11-28

**Authors:** Charilaos Xenodochidis, Kamelia Hristova-Panusheva, Trayana Kamenska, Poornima Budime Santhosh, Todor Petrov, Lyubomir Stoychev, Julia Genova, Natalia Krasteva

**Affiliations:** 1 Institute of Biophysics and Biomedical Engineering, Bulgarian Academy of Sciences, Acad. G. Bonchev Str. Bl.21, 1113 Sofia, Bulgaria; xenodochidis.ch@gmail.com (C.X.); kamelia.t.hristova@gmail.com (K.H.-P.); trayanakamenska@abv.bg (T.K.); 2 Institute of Solid State Physics, Bulgarian Academy of Sciences, Tzarigradsko Chaussee 72, 1784 Sofia, Bulgaria; poorni@issp.bas.bg (P.B.S.); tspetrov@issp.bas.bg (T.P.); lyubomir.stoychev@gmail.com (L.S.); ulia@issp.bas.bg (J.G.); 3 Faculty of Applied Mathematics and Informatics, Technical University of Sofia, 8 Kliment Ohridski Str., 1000 Sofia, Bulgaria

**Keywords:** 515 nm light, 1030 nm light, pulse laser, cell viability, HepG2 cells, MDCK cells

## Abstract

Graphene oxide-mediated photothermal therapy using femtosecond lasers has recently shown promise in treating hepatocellular carcinoma. However, significant work remains to optimize irradiation parameters for specific nanoparticle types and cancer cells to improve nanomaterial-mediated photothermal anticancer therapy. This study investigated the photothermal potential of nGO and nGO-PEG nanoparticles (NPs) combined with femtosecond laser irradiation at 515 nm and 1030 nm wavelengths, with varying power (0.1 and 0.2 W/cm^2^) and duration (5 and 10 min), to optimize photothermal therapy for hepatocellular carcinoma. Conversion efficiency of NPs, morphology and viability of HepG2 and normal MDCK cells after treatments were evaluated using an electronic thermometer, phase-contrast microscopy, and WST-1 assay. The results revealed that nGO-PEG NPs exhibited better photothermal efficiency than nGO, with 515 nm of irradiation inducing a temperature increase up to 19.1 °C compared to 4.7 °C with 1030 nm of light. Laser exposure to 515 nm significantly reduced HepG2 cell viability, with the most intense conditions (10 min at 0.2 W/cm^2^) causing a decrease of up to 58.2% with nGO and 43.51% with nGO-PEG. Normal MDCK cells showed minimal impact or a slight viability increase, especially with nGO-PEG. Combined treatment with laser irradiation and NPs induced significant morphological changes in HepG2 cells, including cell detachment and apoptotic-like characteristics, particularly with 1030 nm of irradiation. MDCK cells exhibited minimal morphological changes, with some recovery observed under lower energy conditions. These findings suggest that low-energy lasers and engineered nanomaterials could provide a minimally invasive approach to photothermal cancer therapy with reduced side effects.

## 1. Introduction

On a global scale, liver cancer has become a major burden impacting social, financial, and healthcare sectors. It ranks among the most lethal diseases, and experts predict a 55% increase in mortality rates by 2040 compared to 2020 worldwide [1]. Hepatocellular carcinoma (HCC) is the most common primary liver tumour, as 85% of HCC cases occur in patients with cirrhosis [2]. These alarming statistics underscore the urgent need to discover or optimize therapeutic approaches for HCC. Diagnostic and treatment approaches vary based on the stage of the disease [3]. Diagnosing early-stage HCC, however, remains challenging, while treatment options for advanced HCC are limited and often associated with severe side effects and toxicity, reducing their overall effectiveness [4,5,6].

Photo-thermal therapy (PTT) has recently gained attention as a prospective method for tumour treatment because it is less invasive, less toxic, and cost-effective [7]. In PTT, photosensitizing agents are activated under electromagnetic radiation, such as radio frequency, microwaves, near-infrared (NIR), or visible light, converting light energy into heat [8,9]. The generated heat causes hyperthermia which can lead to irreversible damage to proteins and DNA, and consequently to tumour cell death [10]. Unlike traditional hyperthermia which produces a maximum temperature gradient on the body surface, and most of the energy dissipates in healthy tissue in the external radiation path, nanomaterial-mediated hyperthermia can concentrate the power of the light on tumours to induce tumour death [11]. Thus, local thermal destruction minimizes the adverse impacts on healthy tissue and allows for precise and on-demand treatments. Additionally, a controlled temperature increase can encourage tissue regeneration, stimulate drug release, boost drug delivery effectiveness, alleviate hypoxia, and enhance other therapeutic processes. These benefits further support the combination of PTT with other treatment approaches, aiming for improved therapeutic outcomes through additive or synergistic effects [12,13,14,15].

Numerous photothermal agents have been extensively studied to enhance the photothermal conversion efficiency, overcome the disadvantage of inhomogeneous heat distribution, and thereby improve the thermal lethality of subcutaneous tumours. Some of these agents have been used for clinical research [11,16,17]. The most widely used nanomaterials for PTT are those with strong absorbance in the NIR region (700–1000 nm). Currently developed photothermal agents mainly include noble-metal nanostructures, transition-metal chalcogenides, C-based materials, and organic materials [11]. C-based materials include single-walled C nanotubes (CNTs), graphene, graphene oxide (GO), and reduced GO [18,19]. Recently, there has been a growing interest in using GO, a graphene derivative, for the photothermal therapy of cancer and other diseases. Due to its unique properties, including a 2D structure with a diameter of 100–300 nm [20] and high NIR absorbance in the I (650–950 nm) and II (1000–1350 nm) biological windows, GO and more specifically reduced graphene oxide have been of particular interest when a continuous wave laser is used [21,22]. To avoid excessive heating obtained under continuous wave laser irradiation, laser energy is often delivered in a pulsed manner, with intervals between pulses exceeding the tissue’s inherent thermal relaxation time. The pulsed laser irradiation at NIR is the most commonly used energy source in PTT, as it delivers high amounts of power in short bursts, allowing normal tissue to recover during extended intervals [22,23].

Femtosecond (fs) pulsed lasers represent an advanced tool that offers precision and the control of emitted electromagnetic radiation compared to nano and picosecond lasers [24]. By generating ultra-short pulses in 10–15 s, fs lasers can rapidly heat tumour cells, leading to their destruction [25]. However, the high amount of energy in these laser pulses can raise the temperature of noble nanoparticles, such as gold NPs, to their melting point, potentially altering their properties. This has prompted significant interest in graphene-based nanoparticles for PTT. For example, Syama and Mohanan reported that combining fs laser irradiation with reduced graphene oxide (rGO) effectively enhances cancer cell destruction through a microcavitation effect [26]. Despite the promising findings from previous studies, a significant research gap remains regarding the optimization of parameters of fs laser irradiation combined with GO-based nanoparticles, across different cell models. This study aims to address that gap by investigating the effects of low-intensity fs laser irradiation with varying parameters, such as duration and power density, alongside two types of graphene oxide nanoparticles—nGO and nGO-PEG—on two distinct cell lines: the hepatocellular carcinoma cell line HepG2 and the normal Madin-Darby canine kidney (MDCK) cell line. The HepG2 cell line is a widely used in vitro model for HCC research due to its clinical relevance and biological characteristics. It retains key features of hepatocyte biology, including metabolic activity, making it suitable for evaluating therapeutic strategies such as photothermal therapy. Using HepG2 cells allows us to investigate the efficacy of graphene oxide nanoparticles in a model that closely reflects the challenges faced in treating liver cancer. Given that different types of light interact with biological tissues in unique ways, we have used two wavelengths as a laser source, 515 nm (green light) and 1030 nm (near-infrared, NIR). NIR light, especially within the NIR-II window (1000–1700 nm), is known for superior tissue penetration and minimal absorption by biological tissues, making it ideal for PTT. While visible and UV light offer less penetration, they can still effectively target and destroy cancer cells. Thus, the impact of various irradiation parameters on the photoconversation potential of nanoparticles and the viability and morphology of both cell types were studied to optimize conditions for enhanced outcomes in photothermal anticancer therapy. Additionally, the potential of this combination as a treatment for hepatocellular carcinoma was explored.

## 2. Results

### 2.1. Characterization of nGO and nGO-PEG NPs

**Figure 1:**
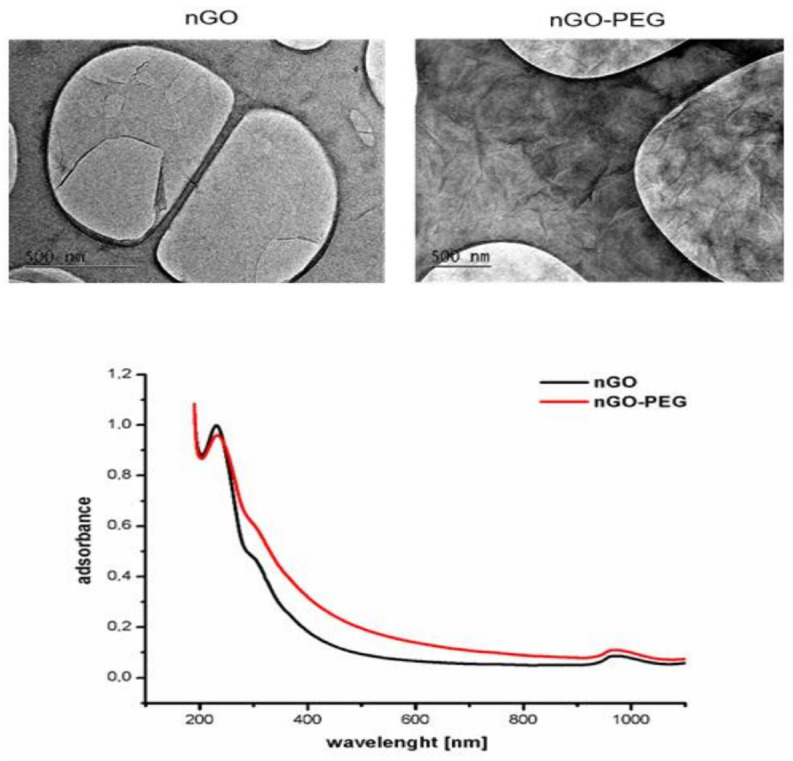
Physiochemical properties of nGO and nGO-PEG NPs: TEM analysis of nGO (**left**) and nGO-PEG after sonication (**right**); UV-Vis adsorbance spectra of nGO and nGO-PEG non-irradiated.

Different techniques, including TEM, UV-Vis spectroscopy, and DLS, were used to characterize the NPs. TEM images showed that nGO had a sheet-like morphology with a rough surface while nGO-PEG exhibited a wrinkled structure, likely due to the presence of PEG at the sheet edges (Figure 1 left). The average sizes of nGO and nGO-PEG were 273.6 nm and 394.2 nm, respectively. Both nanoparticles had negative ζ-potential values, measured at −18.32 mV for nGO and −15.07 mV for nGO-PEG (Table 1). It is generally accepted that in the range of −30 ÷ 30 mV, the NPs’ stability is considered high due to fewer Van Der Waals interparticle attractions [27]. nGO-PEG was less negatively charged in a colloidal system than the pristine nGO particles. PEG probably ensured steric stability by providing a physical barrier that prevented particle aggregation. Subsequently, both NPs had a ζ-potential within the range of stability, thus we have proceeded with an evaluation of cell viability and metabolic activity.

We further measured the UV-Vis spectra of the nGO and nGO-PEG NPs (Figure 1 right) to investigate the NPs’ absorbance in the range of 190–1100 nm, covering the UV-Vis-NIR region of the electromagnetic spectrum.

A peak in absorbance was observed at 233 nm for nGO-PEG, which was slightly higher than the 230 nm peak for nGO. When comparing the absorbance of the nanoparticles at the wavelengths of 515 nm and 1030 nm, nGO-PEG demonstrated greater absorbance than nGO at both wavelengths. Additionally, both nanoparticles showed higher absorbance in green light (515 nm) compared to near-infrared light (1030 nm).

### 2.2. Laser-Induced Temperature Increase In Vitro

We explored the changes in temperature of the cell culture medium (DMEM) with 10% FBS, containing NPs before and after irradiation because the temperature rise is an important characteristic of the NPs’ photothermal ability and their potential as photothermal agents (PAs) (Table 2 and Table 3).

The temperature measurements of DMEM with and without nanoparticles under laser irradiation at a power density (PD) of 0.1 W/cm^2^ are summarized in Table 2. Green light (515 nm) irradiation increased the medium’s temperature by 5.4 °C after 5 min and 7.8 °C after 10 min. In DMEM containing nGO, the temperature increased by 7.0 °C after 5 min and by 9.7 °C after 10 min. The temperature rise was proportionally pronounced in DMEM containing nGO-PEG, with an increase of 7.4 °C after 5 min and 10.2 °C after 10 min of green light exposure. On the other hand, NIR light induced a temperature rise of 0.6 °C after 5 min and 1.2 °C after 10 min of exposure of the buffer. Similar rises observed in the solution after the addition of nGO, where the increase was 1.1 °C after 5 min and 1.7 °C after 10 min of exposure. The temperature increase in the medium with nGO-PEG was the highest: 2.4 °C and 3.1 °C after 5 min and 10 min of exposure, respectively.

Table 3 presents data on temperature changes in buffer solutions with and without nanoparticles after 5 and 10 min of exposure to electromagnetic radiation at 515 nm and 1030 nm, with the power density of the fs laser being 0.2 W/cm^2^.

In DMEM without NPs, a temperature increase of 10.1 °C was detected after 5 min of green light irradiation, with a further rise to 13.7 °C after 10 min. Conversely, NIR light resulted in a 1.8 °C increase after 5 min and a 2.4 °C increase after 10 min. Irradiation of DMEM containing nGO with green light (515 nm) significantly altered the temperature after 5 min with the temperature rises of 12.1 °C and 17.3 °C after 5 and 10 min of exposure, respectively. However, the temperature rise in the DMEM with nGO after NIR irradiation was significantly modest: 2.6 °C and 3.3 °C after 5 and 10 min of exposure, respectively. A similar tendency was detected in the DMEM containing nGO-PEG NPs, with higher temperature increases of 14.3 °C and 19.1 °C after 5 and 10 min of exposure to green light, and a 3.5 °C rise after 5 min and a 4.7 °C increase after 10 min of exposure to NIR light. Overall, the results indicate that the 0.2 W/cm^2^ fs laser caused greater temperature elevations across all solutions, demonstrating a clear correlation between higher PD and increased temperature in both nanoparticle-containing and nanoparticle-free solutions.

### 2.3. Synergistic Effect of Laser Irradiation and NPs on MDCK and HepG2 Cell Viability

**Figure 2:**
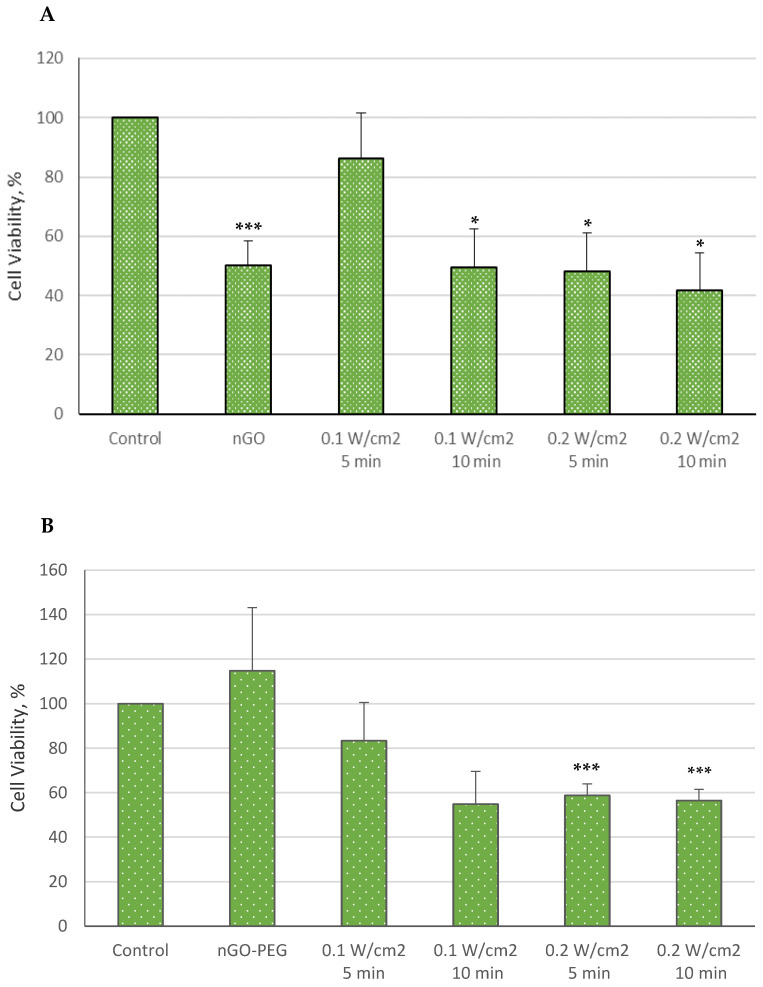
Cell viability of HepG2 cells treated with nGO (**A**) and nGO-PEG NPs (**B**) with a concentration of 100 μg/mL, irradiated with a laser beam at 515 nm for 5 and 10 min and with power densities of 0.1 and 0.2 W/cm^2^. The results’ statistical significance was denoted as * *p* < 0.05; *** *p* < 0.005.

**Figure 3:**
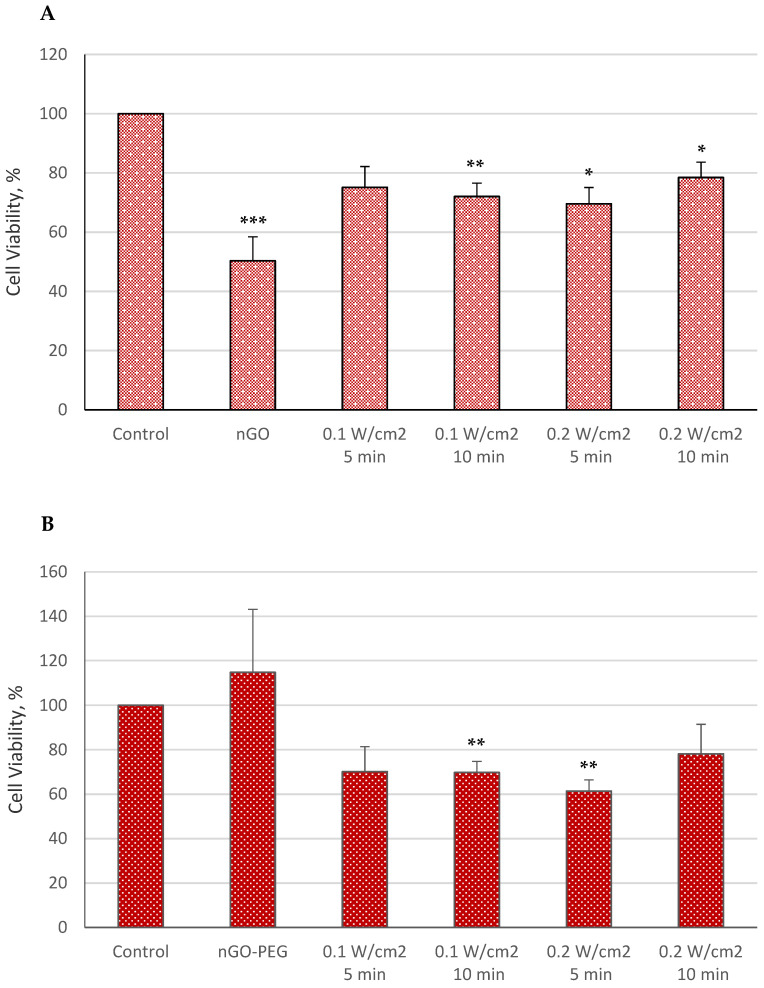
Cell viability of HepG2 cells treated with nGO (**A**) and nGO-PEG NPs (**B**) with a concentration of 100 μg/mL, irradiated with a laser beam at 1030 nm for 5 and 10 min and with power densities of 0.1 and 0.2 W/cm^2^. The results’ statistical significance was denoted as * *p* < 0.05, ** *p* < 0.01, and *** *p* < 0.005.

We conducted a biological evaluation of the combined effects of nGO and nGO-PEG nanoparticles with femtosecond (fs) laser irradiation on the hepatocellular cell line HepG2, in comparison to normal MDCK cells, assessing both viability and morphology. The viability results for HepG2 cells are presented in Figure 2 and Figure 3

As shown in Figure 2A, the combination of nGO or nGO-PEG with laser irradiation at 515 nm and a power density of 0.1 W/cm^2^ significantly reduced cell viability compared to the control group (untreated and non-irradiated cells). The decrease in HepG2 cell viability following treatment with nGO NPs ranged from 13.77% to 58.2%, with the greatest reduction observed under the most intense irradiation conditions (10 min at 0.2 W/cm^2^). In contrast, the mildest effect was seen with 5 min of irradiation at 0.1 W/cm^2^.

A similar trend was observed with nGO-PEG NPs combined with irradiation at 515 nm and 0.1 W/cm^2^, though the reduction in viability was slightly less pronounced than that with nGO NPs, ranging from 16.61% to 43.51% (Figure 2B). The smallest decrease in metabolic activity occurred in cells irradiated for 5 min at 0.1 W/cm^2^, while differences among the other irradiated groups were minimal. Interestingly, treatment with nGO-PEG alone resulted in an approximately 15% increase in cell viability compared to the control cells, in contrast to the nGO NP treatment, although this result was not statistically significant.

Additionally, combining 1030 nm of irradiation with nGO and nGO-PEG nanoparticles led to a decrease in cell viability, though the effect was less pronounced than with 515 nm of irradiation. The outcomes across various 1030 nm irradiation groups were relatively similar, suggesting an absence of a clear linear correlation between physical parameters (such as exposure time and power density) and HepG2 cell viability (Figure 3A), likely due to the modest temperature increase of the medium.

**Figure 4:**
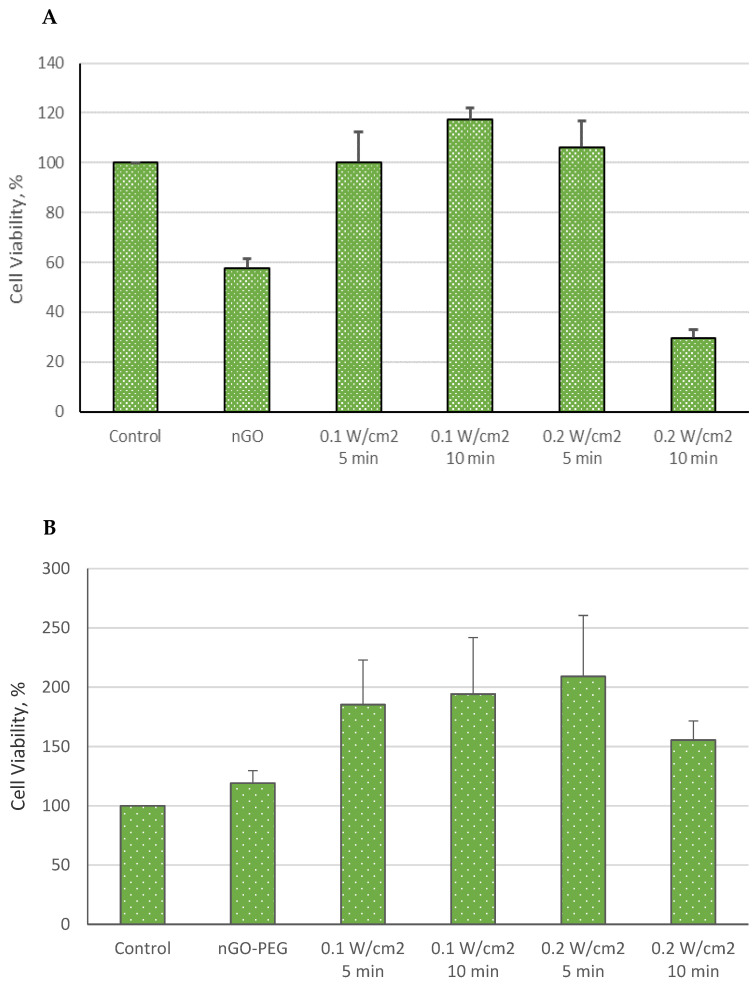
Cell viability of MDCK cells treated with nGO (**A**) and nGO-PEG NPs (**B**) with a concentration of 100 μg/mL, irradiated with a laser beam at 515 nm for 5 and 10 min and with power densities of 0.1 and 0.2 W/cm^2^.

**Figure 5:**
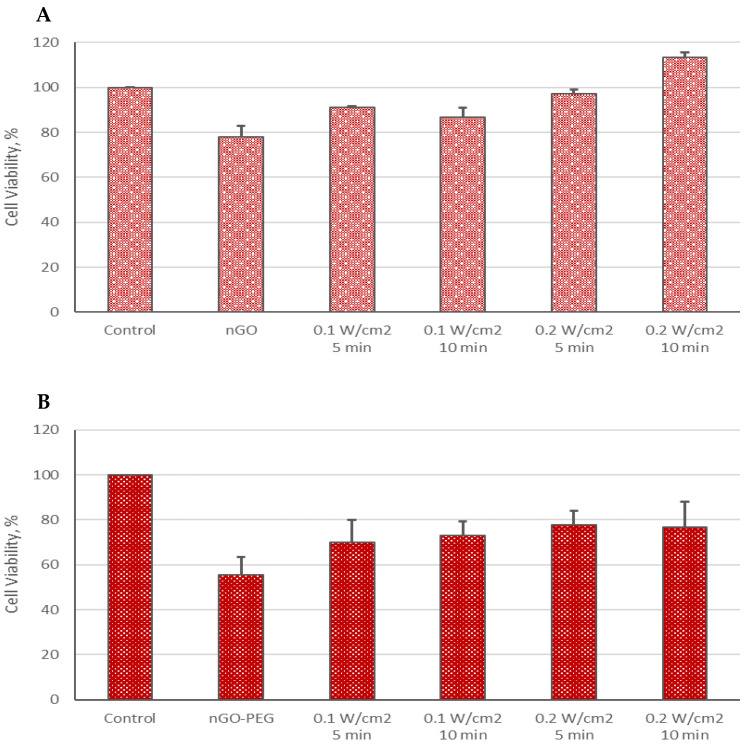
Cell viability of MDCK cells treated with nGO (**A**) and nGO-PEG NPs (**B**) with a concentration of 100 μg/mL, irradiated with a laser beam at 1030 nm for 5 and 10 min and with power densities of 0.1 and 0.2 W/cm^2^.

The effects of nGO and nGO-PEG NPs, both independently and in combination with laser irradiation, on the viability of normal MDCK cells are depicted in Figure 4 and Figure 5.

As shown in Figure 4A, treatment with nGO alone reduced MDCK cell viability by 41.4% compared to the control group. Interestingly, when MDCK cells were simultaneously treated with nGO NPs and 515 nm of laser irradiation at 0.1 W/cm^2^ for 5 min, there was no significant impact on cell viability. However, extending the irradiation to 10 min at the same power density and increasing the power density to 0.2 W/cm^2^ for 5 min resulted in a stimulatory effect on cell viability compared to controls. Contrarily, a 10 min irradiation at 0.2 W/cm^2^ significantly inhibited cell viability, reducing it by up to 70%. Despite these observations, *t*-test analysis did not reveal statistically significant differences in MDCK cell viability between the treated and control groups.

Similarly, Figure 4B presents the changes in MDCK cell viability following treatment with nGO-PEG NPs. Notably, nGO-PEG treatment increased MDCK cell viability across all groups. When combined with 515 nm of laser irradiation, nGO-PEG NPs further stimulated MDCK metabolic activity and cell viability. In this scenario, all tested irradiation parameters enhanced cell viability compared to the control group. The most pronounced synergistic effects were observed in cells treated at 0.1 W/cm^2^ for 5 and 10 min, as well as those irradiated at 0.2 W/cm^2^ for 5 min. The least pronounced effect was seen in cells irradiated for 10 min at 0.2 W/cm^2^.

Figure 5 presents the results of MDCK cell viability following irradiation with a 1030 nm laser beam. When combined with nGO NPs, the 1030 nm of irradiation produced only a slight inhibitory effect on cell viability, with reductions ranging from 1.3% to 7.5% compared to the control cells. Interestingly, under the strongest irradiation conditions—10 min at 0.2 W/cm^2^—there was a 15% increase in cell viability (Figure 5A). However, none of these changes in MDCK cell viability were statistically significant compared to the control group. In contrast, when nGO-PEG NPs were combined with 1030 nm of irradiation, a more pronounced decrease in cell viability was observed, ranging from 22% to 29% compared to the control group. Variations in exposure time and power density had minimal impact on these results (Figure 5B). Notably, treatment with nGO-PEG NPs alone also reduced MDCK cell viability, similar to the effect seen with nGO NPs. However, in HepG2 cells, nGO-PEG NPs exhibited a stimulatory effect on cell viability, in contrast to their inhibitory effect on MDCK cells.

### 2.4. Synergistic Effect of Laser Irradiation and NPs on MDCK and HepG2 Cell Morphology

**Figure 6:**
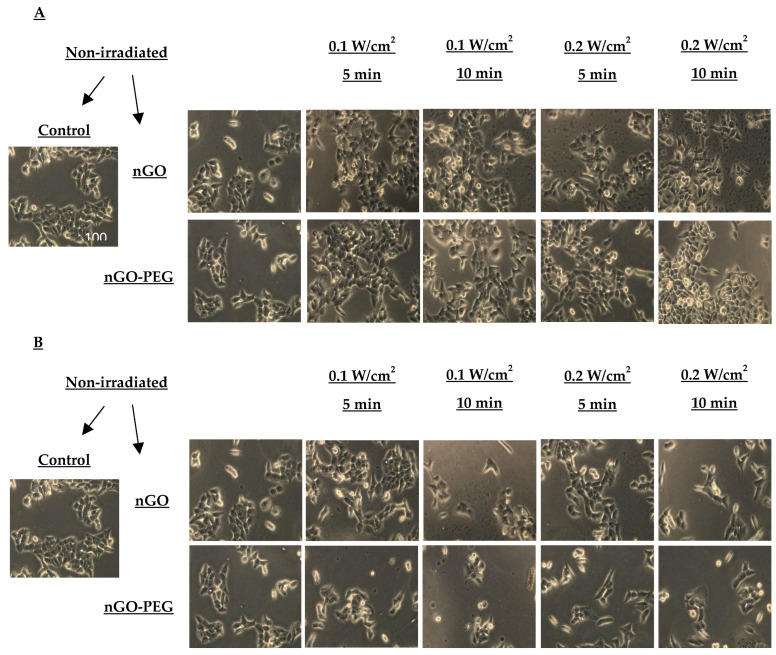
Morphological changes in the HepG2 cell line treated with nGO and nGO-PEG NPs with a concentration of 100 μg/mL a day after irradiation with a laser beam at 515 nm (**A**) and 1030 nm (**B**) for 5 and 10 min and with power densities of 0.1 and 0.2 W/cm^2^.

To confirm the anticancer activity of synergistic treatment combining fs laser irradiation and NPs, we analyzed the overall morphology of hepatocellular HepG2 cells and normal MDCK cells using phase-contrast light microscopy after 24 h of exposure. Figure 6 illustrates the morphological changes in HepG2 cells non-irradiated and irradiated with a fs laser emitting electromagnetic radiation at 515 nm and 1030 nm, respectively.

As shown in Figure 6, HepG2 cells in the control group displayed a normal polygonal shape, clear boundaries, and were well-spread on the cell substratum. Treatment with nGO and nGO_PEG NPs resulted in a decreased number of attached cells, with more cells exhibiting an elongated or rounded morphology and the presence of microvilli. Combination treatment with both types of NPs and 515 nm of irradiation at 0.1 W/cm^2^ slightly affected the cell morphology of viable cells. The higher energy of 0.2 W/cm^2^ however has a stronger effect, resulting in many cells displaying apoptotic-like characteristics with numerous vacuoles inside. In nGO-PEG-treated cells, the majority of vacuoles were observed in the cell periphery, also suggesting an altered osmotic balance of the cells.

**Figure 7:**
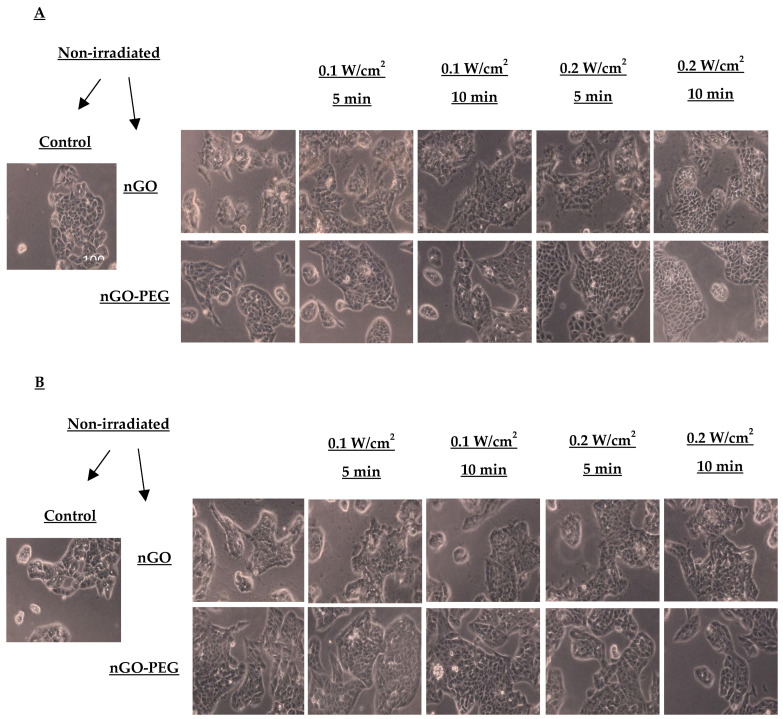
Morphological changes in the MDCK cell line treated with nGO and nGO-PEG NPs with a concentration of 100 μg/mL a day after irradiation with a laser beam at 515 nm (**A**) and 1030 nm (**B**) for 5 and 10 min and with power densities of 0.1 and 0.2 W/cm^2^.

Irradiation of HepG2 cells with 1030 nm laser beams had an even greater impact on cell morphology, significantly reducing the number of attached cells and cell density (Figure 7A). The most pronounced decrease in cell density was observed when cells were irradiated for 10 min with 0.1 W/cm^2^, and for 5 and 10 min with 0.2 W/cm^2^. The cell shapes were predominantly elongated or rounded, with many well-expressed microvilli, indicating a synergistic anticancer effect from the combination of the 1030 nm laser beam and both types of NPs.

Morphological changes in normal MDCK cells 24 h after treatment with NPs and laser irradiation are shown in Figure 7. Control MDCK cells were well-adhered to the substratum, and exhibited a characteristic cobblestone morphology with well-defined cell boundaries. Following exposure to nGO, cells lost cell-to-cell contacts, acquired a more blurred morphology, and formed smaller aggregates.

Synergistic treatment of MDCK with nGO-PEG and 515 nm of irradiation at 0.1 W/cm^2^ and 0.2 W/cm^2^ for 5 min appeared to restore cell morphology. However, 10 min of irradiation resulted in necrotic/apoptotic-like cells with numerous vacuoles. A similar effect was observed after combined treatment with nGO and 10 min of irradiation at 0.2 W/cm^2^. Other combinations of nGO and 515 nm irradiation parameters only slightly affected cell morphology.

When MDCK cells were treated with NPs and irradiated with 1030 nm laser beams, no significant changes in cell morphology were detected. Only a slight cell swelling was observed with the combination of nGO NPs and 1030 nm of irradiation. It appears that healthy cells were not significantly affected by the application of a femtosecond laser and its synergy with NPs.

## 3. Discussion

Here, we reported on the photothermal effects of nGO and nGO-PEG nanoparticles in hepatocellular carcinoma and normal cells after irradiation with a low-intensity fs laser at two wavelengths: 515 nm and 1030 nm, while varying the physical parameters of the light. The primary focus of our experiments involving cellular models and temperature measurements was on NIR irradiation because NIR light is far more practical for clinical applications than green light due to its superior penetration depth and reduced scattering in biological tissues. In this study, we used green laser irradiation as a comparative tool to evaluate the optical and photothermal properties of the nanoparticles under different wavelengths.

Although GO and GO-PEG NPs have been previously explored as photothermal agents [28,29,30], there are still numerous challenges that need to be addressed before their clinical use. Reduced GO NPs are often considered to be among the most promising types of GO for photothermal therapy due to their excellent absorption properties, especially in the NIR range [31,32]. However, their synthesis is frequently associated with toxic byproducts, which limits their biomedical applications. To overcome these limitations, the nGO-PEG used in this work was obtained by a simple and fast method of simultaneous reduction and PEGylation of GO. This approach results in no toxic waste and in increased absorption in the UV-Vis and NIR regions confirmed by the physicochemical characterization of both NPs. A thorough description of the preparation of nGO and nGO-PEG NPs and their physico-chemical characterization has been performed in our previous publications [33,34].

In this study, we first investigated the photothermal abilities of the nanoparticles by measuring the temperature increase in complete cell culture medium (DMEM with 10% FBS) containing nGO and nGO-PEG under laser irradiation at wavelengths of 515 nm and 1030 nm. We found that the temperature increase in the cell culture medium containing nGO-PEG was higher than in those containing nGO, indicating the better photothermal conversion efficiency of nGO-PEG. This suggests that nGO-PEG more effectively absorbs energy from both green and NIR irradiation, converting it into heat more efficiently. This characteristic could be crucial for patients in reducing the time required to reach and maintain the desired temperature in treatment areas. Our findings align with those reported by Malgorzata J. Podolska et al. [35]. They showed that PEGylation slightly impact the photothermal conversion efficiency of rGO, after 1 h of irradiation at 960 nm with a power density of 2 W/cm^2^. However, even if PEGylated GO does not surpass GO in terms of photothermal conversion efficiency, it is still preferable for PTT due to its superior physicochemical properties such as its water dispersity as well as biocompatibility.

Further analysis of photothermal conversion revealed intriguing insights. Green light induced a significantly higher temperature increase in all samples (ranging from 5.4 °C to 19.1 °C), while NIR irradiation resulted in a more modest increase (from 0.6 °C to 4.7 °C). Among the NPs tested, nGO-PEG generated a higher temperature, approximately 2 °C higher than that of nGO. Notably, varying the irradiation parameters showed that a power density in the studied range of 0.1–0.2 W/cm^2^ at 1030 nm had a similar photothermal effect on DMEM with both types of NPs, while that at 515 nm 0.2 W/cm^2^ induced a higher temperature increase in DMEM containing both types of NPs than 0.1 W/cm^2^ did. Increasing the duration of irradiation from 5 to 10 min led to a more pronounced temperature rise at both wavelengths. While the study explores two laser wavelengths, power densities, and irradiation durations, further optimization of these parameters could improve therapeutic efficacy. The optimal conditions for different tumour types or patient conditions may differ and require more extensive parameter optimization.

It is crucial to consider that the initial temperature in these measurements was room temperature, around 23–25 °C. In a human body context, the temperature increase under NIR irradiation would likely reach 42–45 °C, while under green light, it could range from 54.1 °C to 72.9 °C. Studies showed that tumour temperatures of 43–50 °C promote apoptosis, while exceeding 50 °C leads to necrosis [36,37,38,39,40]. Our findings which demonstrate that the temperatures remained under 50 °C, suggest apoptosis as the main mechanism, which is preferable for cell death. However, we also have to keep in mind that the results may not fully translate to in vivo systems, where factors like tissue penetration, immune response, and nanoparticle distribution may significantly differ. We further evaluated cell morphology and viability alterations to understand if the combination of GOs NPs and fs laser irradiation is a suitable approach for hepatocellular cancer treatment. The greatest reduction in HepG2 cell viability was observed with the combination of NPs and laser irradiation at 515 nm. Moreover, the most substantial decrease was detected under the strongest irradiation conditions (10 min at 0.2 W/cm^2^). The reduction in cell viability ranged from 13.77% to 58.2% for nGO and 16.61% to 43.51% for nGO-PEG. This indicated a synergistic effect of laser irradiation and NPs, with nGO-PEG showing a slightly less potent but still significant inhibitory effect on cancer cell viability. However, this finding was in contrast with the results obtained from photocamera measurements. To our knowledge, no findings support such an efficiency of the green light in hepatocellular cancer treatment. The latter will be the focal point of our future investigations.

Interestingly, the effects of the NPs on normal MDCK cells were markedly different. Treatment with nGO alone decreased MDCK cell viability, but when combined with 515 nm of laser irradiation, some irradiation parameters even have a stimulatory effect on cell viability, especially when combined with nGO-PEG. This differential impact highlights the potential of these NPs to selectively target cancer cells while sparing or even promoting the viability of normal cells, which is a desirable trait in cancer therapies. One explanation for the observed effects could be the difference in how cancerous and normal cells process and respond to NPs. For instance, in cancer cells, NPs often induce oxidative stress through the generation of reactive oxygen species (ROS), leading to cell damage and death. In contrast, normal cells might better manage oxidative stress because their antioxidant mechanisms are not damaged. Our assumption for the observed stimulatory effect in MDCK cells upon nGO and especially nGO-PEG NPs and laser treatment could be related to mild stress-induced proliferation, a phenomenon where low levels of stress trigger repair and growth pathways in healthy cells [41]. This enables normal cells to either survive or even proliferate under conditions where cancer cells perish [42]. Studies on gold nanoparticles have shown similar selective effects where cancer cells undergo apoptosis due to mitochondrial damage [43,44,45], while normal cells maintain their viability by resisting oxidative stress. In some cases, mild oxidative stress induced by nanoparticles might also temporarily increase the metabolic activity of cancer cells, leading to the apparent stimulation of cell viability. One possibility is that graphene oxide’s ROS generated at lower levels may act as secondary messengers, triggering pro-survival signalling pathways. Therefore, the observed stimulatory effect of nGO-PEG on HepG2 cell viability under certain conditions could be attributed to several factors, including the nanoparticles’ interaction with the cellular microenvironment.

The morphological analysis of HepG2 and MDCK cells after treatment with NPs and laser irradiation further confirmed the selective cytotoxicity of the treatment. Under the influence of stronger irradiation, namely 0.2 W/cm^2^, HepG2 cells showed significant morphological changes indicative of apoptosis, particularly when combined with nGO-PEG. The obtained data confirmed the decreased cell viability and metabolic activity after the synergic effect of light and NPs. This discovery may be due to the production of ROS. Generally, apoptotic-like behaviour results from induced oxidative stress, which can be enhanced by laser irradiation and NPs. This hypothesis is based on a similar report by Grześkowiak and colleagues, who developed nanoparticle-based platforms. The latter, when combined with an NIR laser, showed high photothermal conversion efficacy leading to enhanced cytotoxicity in hepatocellular carcinoma cells [46]. In contrast, MDCK cells generally maintained their normal morphology, even under similar treatment conditions, although some losses of cell-to-cell contacts and blurred morphology were observed.

Overall, these findings suggest that nGO and nGO-PEG, particularly when combined with targeted laser irradiation, hold significant promise for photothermal therapy. The selective cytotoxicity towards cancer cells, combined with the ability to maintain normal cell viability, underscores the potential of these NPs as effective. However, this study uses only HepG2 (hepatocellular carcinoma) and MDCK (normal) cell lines, which may not represent the full spectrum of cancer and normal cell types. The response to photothermal therapy could vary in different cancer types and healthy tissues. Further, this study focuses on short-term effects (24 h post-treatment) but does not assess the long-term impacts of the treatment, such as cell recovery or potential resistance development. Long-term toxicity and side effects, especially in normal cells, need further investigation.

Also, although this study discusses the potential production of reactive oxygen species (ROS) and apoptosis in HepG2 cells, the precise molecular mechanisms behind the differential effects on normal and cancer cells remain unclear. Future studies should further explore the mechanisms underlying these differential effects and optimize the physical parameters to maximize therapeutic efficacy.

## 4. Materials and Methods

### 4.1. Preparation of nGO and nGO-PEG NPs

Graphene Oxide (C1576, Graphenea, San Sebastián, Spain) was initially provided as water suspensions at a concentration of 4 mg/mL. For in vitro exposures, a stock solution of 1 mg/mL in deionized water was prepared, then sonicated at 500 Hz for 2 h on an ultrasonic homogenizer (VCX 500, Sonics and Materials, Inc., Newtown, CT, USA) to obtain nanosized particles. PEGylation of the nanosized GO (nGO) was performed by simultaneous reduction and modification in a water bath overnight at 70 °C using mPEG-NH_2_ (Abbexa Ltd., Cambridge, UK) as previously described [20]. Immediately before experiments, the nanoparticles were sonicated for an hour in an ultrasonic water bath (40 Hz, UM-2, Unitra-Unima, Olsztyn, Poland). For in vitro experiments, nanoparticle suspensions were prepared at a final concentration of 0.1 mg/mL.

### 4.2. Physico-Chemical Characterization of the NPs

- ζ-potential and particle size analysis

Zetasizer Nano ZS (Malvern Instruments, Malvern, UK) with a detection angle of 173° was used to measure the ζ-potential and size distribution. All measurements were carried out using a laser with a 633 nm wavelength at a constant temperature of 25 °C. The samples were run in DTS1070 Disposable Folded Capillary cell (for Zeta potential)/DTS0012 Plastic (Malvern Instruments, Malvern, UK).

- UV-Vis spectral measurements

The UV-Vis spectral measurements of nGO and nGO-PEG were conducted on a Specord 210 Plus spectrophotometer (Edition 2010, Analytik Jena AG, Jena, Germany).

- Transmission electron microscopy (TEM)

TEM images of nGO and nGO-PEG were captured by employing Holey carbon film on 300 mesh nickel grids and a JEOL TEM (model JEM-2100, Tokyo, Japan), operated at 200 kV.

### 4.3. Cell Line and Cell Culture

HepG2 and MDCK cell lines were cultured in Dulbecco’s modified Eagle’s medium (DMEM) supplemented with 10% fetal bovine serum (FBS; Sigma-Aldrich, Darmstadt, Germany). The cells were grown in a humidified incubator at 37 °C, with 5% CO2 and 95% humidity. To detach the cells for in vitro experiments, a solution containing 0.05% trypsin and 0.02% EDTA (Sigma-Aldrich, Germany) was used. Following detachment, the cells were seeded into 24-well plates at a density of 2.5 × 10^4^ cells per well. After a 24 h cultivation period, the cells were treated with nGO and nGO-PEG nanoparticles in 0.1 mg/mL concentrations and incubated for another 24 h before laser exposure.

### 4.4. Laser Set-Up and Laser-Induced Temperature Increase In Vitro

The laser-induced temperature increase of the buffer (DMEM medium with 10% FBS), the DMEN containing 0.1 mg/mL nGO or nGO-PEG NPs, was measured using a Pharos laser device (Ph2-10-1000-02-H0-B, Light Conversion UAB, Vilnius, Lithuania) with different irradiation powers: 0.1 W/cm^2^ and 0.2 W/cm^2^. Irradiation was performed with bi/burst mode (8 impulses) and an automated harmonic generator, operating at two wavelengths (λ) of 515 and 1030 nm at different power densities (PDs) (W/cm^2^) with maximum output powers of 10 W and 5.9 W, respectively. The operating pulse widths were 170 fs at 1030 nm and 130 fs at 515 nm. The output powers of the laser radiation at all wavelengths were finely attenuated by internal power control. The attenuation for 515 nm was 17.5%; and for the 1030 nm, it was 4.7%. This allowed for setting the appropriate value to have equal power densities on all samples. Laser irradiation was performed 30 min after placing NPs in the wells in 24-well plates. For each power setting of 0.1 and 0.2 W/cm^2^, an exposure time of 30 s was used in the two wavelengths. The real-time temperature was recorded every 10 s with an electronic thermometer.

### 4.5. Experimental Design of In Vitro Cell Culture Experiments

Cells, both treated and untreated with nanoparticles (NPs), were exposed to wavelengths of 515 nm and 1030 nm for 5 and 10 min, at power densities of 0.1 W/cm^2^ and 0.2 W/cm^2^. MDCK and HepG2 cells were categorized into six groups based on the irradiation parameters:

Group 1 (Control): Untreated cells, non-irradiated.
Group 2: Cells treated with nGO or nGO-PEG NPs, non-irradiated.
Group 3: Cells treated with NPs, followed by 5 min of irradiation at 0.1 W/cm^2^.
Group 4: Cells treated with NPs, followed by 10 min of irradiation at 0.1 W/cm^2^.
Group 5: Cells treated with NPs, followed by 5 min of irradiation at 0.2 W/cm^2^.
Group 6: Cells treated with NPs, followed by 10 min of irradiation at 0.2 W/cm^2^.

### 4.6. Phase-Contrast Light Microscopy

Following a 24 h exposure to the tested NPs and laser irradiation, phase-contrast light observations were made to assess changes in cell morphology. Micrographs were taken at magnifications of 10× with a Zeiss microscope (Axiovert 25, Carl Zeiss, Jena, Germany), equipped with a CCD camera.

### 4.7. WST-1 Assay

WST-1 was performed after the irradiation of cells to evaluate metabolic activity and cell viability, correspondingly. Briefly, immediately after irradiation, the medium with NPs was removed, and the cells were washed with PBS. Finally, a 300 μL medium with 30 μL of WST-1 (tetrazolium salt 4-(3-(4-iodophenyl)-2-(4-nitrophenyl)-2 h-5-tetrazolium)-1,3-benzene disulfonate) was added to all wells. Following a 1 h incubation period in the dark, an ELISA reader Thermo Scientific Multiskan Spectrum (Thermo Scientific, Tokyo, Japan) was employed to estimate the optical density of the samples at 450 nm. Cell viability was calculated as follows:

Cell viability (%) = (OD_450_ sample/OD_450_ control) × 100

### 4.8. Statistical Analysis

The obtained data were analyzed using Microsoft Excel software version 10 (Microsoft, Redmond, WA, USA). The presented bars illustrated MEAN values of the MDCK and HepG2 cell viability/metabolic activity ± STDV. Probability levels of * *p* < 0.05, ** *p* < 0.01, and *** *p* < 0.005 were considered statistically significant after the performance of Student’s *t*-test.

## 5. Conclusions

In conclusion, this paper examines the effects of two distinct light wavelengths—515 nm and 1030 nm—along with varying light parameters, in combination with nGO and PEGylated nGO nanoparticles, on the viability and morphology of HepG2 and MDCK cells. Our findings highlight the promising potential of nGO and nGO-PEG NPs in PTT, especially when combined with laser irradiation. Among the two fs laser light sources, green laser light (515 nm) in combination with both NPs showed a greater impact on both cell types, likely due to a significant temperature increase in the solutions with and without NPs. Specifically, the viability of cancerous HepG2 cells was significantly reduced, while in the case of normal MDCK cells, metabolic activity and cell viability were increased. The only exception was the combination of nGO with a higher power density of 0.2 W/cm^2^ at 515 nm, which led to a marked decrease in MDCK cell viability. On the other hand, NIR light (1030 nm) in synergy with nGO and nGO-PEG NPs produced a similar but less pronounced effect on the viability of HepG2 cells. In contrast, NIR light negatively affected the viability of normal MDCK cells, which is opposite to the response observed with green light.

Further research is needed to optimize the physical parameters of NP-laser systems to enhance their therapeutic efficacy. Additionally, understanding the mechanisms behind the selective cytotoxicity and differential cell responses will be crucial for advancing the clinical application of nGO and nGO-PEG combined with laser irradiation. Overall, this study provides valuable insights into the potential of NP-based platforms for targeted cancer therapy, laying the groundwork for further exploration of their clinical utility.

## Figures and Tables

**Table 1:** ζ-potential, polarity and average size of nGO and nGO-PEG in water solution.

NPs	ζ-Potential, mV	Polarity	Average Size, nm
nGO	−18.32	Negative	273.5
nGO-PEG	−15.07	Negative	394.2

**Table 2:** Temperature measurement by the infrared thermometer of the DMEM medium with 10% FBS (buffer) containing nGO and nGO-PEG NPs irradiated for 5 and 10 min with 0.1 W/cm^2^ laser power density at 515 and 1030 nm. Data are presented as the means of three identical measurements.

Wavelength	Buffer			nGO			nGO-PEG		
	0 min	5 min	10 min	0 min	5 min	10 min	0 min	5 min	10 min
515 nm	24.7 °C	30.1 °C	32.5 °C	24.5 °C	31.5 °C	34.2 °C	24.5 °C	31.9 °C	34.7 °C
1030 nm	25.3 °C	25.9 °C	26.5 °C	25.2 °C	26.3 °C	26.9 °C	24.1 °C	26.5 °C	27.2 °C

**Table 3:** Temperature measurement by the infrared thermometer of the DMEM medium with 10% FBS (Buffer) containing nGO and nGO-PEG NPs irradiated for 5 and 10 min with 0.2 W/cm^2^ laser power density at 515 and 1030 nm. Data are presented as the means of three identical measurements.

Wavelength	Buffer			nGO			nGO-PEG		
	0 min	5 min	10 min	0 min	5 min	10 min	0 min	5 min	10 min
515 nm	25.0 °C	35.1 °C	38.7 °C	24.4 °C	36.5 °C	41.7 °C	24.3 °C	38.6 °C	43.4 °C
1030 nm	24.9 °C	26.7 °C	27.3 °C	24.4 °C	27.0 °C	27.7 °C	23.4 °C	26.9 °C	28.1 °C

## Data Availability

The data presented in this study are available on request from the corresponding author.
